# A virtual reality test to evaluate dismounted soldiers’ cognitive and psychomotor performance in an operationally relevant setting

**DOI:** 10.3389/fpsyg.2025.1540936

**Published:** 2026-01-26

**Authors:** Matthijs Koedijk, Annemarie Landman, Charelle Bottenheft, Yvonne M. Fonken, Olaf Binsch

**Affiliations:** 1 TNO Human Factors Research Institute, Soesterberg, Netherlands; 2 Health Department of the Royal Netherlands Army, Netherlands Ministry of Defense, Utrecht, Netherlands

**Keywords:** MOUT, simulation, soldier performance, decision-making, workload, stress, cognition, assessment

## Abstract

**Background:**

Validated and reliable tests are necessary to evaluate the effectiveness of, for example, (data-driven) selection, education and training, and human enhancement interventions, in high-risk professions like the military. Therefore, the current paper describes the development of a test for measuring dismounted soldiers’ ability to make quick decisions in unpredictable close-quarters battle (room-clearing) contexts.

**Methods:**

A group of 15 Dutch Marines Special Operation Forces (SOF) operators and 17 Dutch Army SOF-support specialists individually performed a test in Virtual Reality (VR). Participants could physically walk, shoot, and verbally communicate with opponents. Fifteen performance items indicative of situational awareness (SA) of threats were scored by subject-matter experts, shoot/do not shoot errors were counted, and visual response times (VRTs) in shooting were obtained. Eight room-clearing scenarios were performed twice: once before (pretest) and once following a night of sleep deprivation (posttest), to measure test–retest reliability and sensitivity to a typical military stressor.

**Results:**

The SA items demonstrated adequate internal consistency, and there was a significant test–retest correlation. SOF operators had significantly higher SA item scores (*p* < 0.05) and made fewer shoot/do not shoot errors (*p* < 0.05) than SOF-support specialists. VRTs showed no significant test–retest correlation or effects. Sleep deprivation had no significant effect on any of the performance measures.

**Conclusion:**

The developed test methodology offers a means to obtain embedded measures of SA, as well as shoot/do not shoot decisions, but VRT measurement appeared to be unreliable. Performance measures were not sensitive to effects of sleep deprivation, possibly due to a counteracting learning effect and limitations in timing of the post-test. The reliability checks of the SA measures were promising, indicating that this study contributes to advancing methodologies for evaluating human enhancement interventions on performance in operationally relevant settings. It is advised to incorporate team performance measures to enhance realism and integrate digitally obtained metrics to minimize observer bias.

## 1 Introduction

To evaluate the effectiveness of selection, training, and human enhancement interventions on operational performance, it is important to use reliable and valid tests that reflect real-world operational contexts (Araujo et al., 2007; Di Nota and Huhta, 2019; Staller et al., 2017). Current evaluation methods often rely on simplistic or global measures, such as situational awareness ratings, decision-making assessments, or mission success outcomes (Koedijk et al., 2021). Though straight-forward, these methods often provide limited insights into specific effects of interventions on different facets of performance (Di Nota et al., 2021). To address these limitations, it is essential to expand and validate tests for assessing operational performance. This begins with the development of ecologically-relevant yet controlled tasks, which challenge aspects of human performance. Ideally, applied cognitive performance, such as perceiving different action possibilities in the environment and selecting optimal courses of action based on available information, is assessed using “embedded measures.” These measures are derived from the natural actions that operators take while performing their tasks (Kintz et al., 2023). To elicit natural behaviour, the test environment should expose operators to a variety of realistic (task) constraints that shape and inform their actions (Pinder et al., 2011; Araujo et al., 2007). In previous work, operational evaluation methods using embedded (behavioral) measures typically involve the development of test environments designed to elicit specific, natural behaviours and the creation of appropriate scoring and interpretation frameworks (see, e.g., Baldwin et al., 2022; Bertilsson et al., 2019; Huhta et al., 2021; Koedijk et al., 2021).

The current paper describes the development of a test aimed at measuring aspects of soldiers’ cognitive and psychomotor performance, using embedded performance measures within an operationally-relevant room-clearing task. This test was created from a special operation forces (SOF) perspective, as it was focused on aspects of performance that encompass the quick obtaining of situational awareness (SA), swift decision-making and fast movement execution. This process of perceptual-motor performance is an interactive loop of processing sensory information presented in the environment (perception), interpreting the situation, and selecting a possible action (selection), and finally realizing and initiating actual movements action; (Nieuwenhuys and Oudejans, 2012, 2017; Pijpers et al., 2006). One’s aptitude to maintain a high level of such performance under high pressure is also known as “action-intelligence” in the Dutch SOF community, and is an important factor in SOF selection and training. A room-clearing task with complex room layouts or obstacles and dynamic opponents is particularly suited to challenge these performance components. It features a complex, unpredictable environment with different potential threats, such as blind spots, doors and windows, and areas of vulnerability (i.e., “fatal funnel”), creating high-pressure conditions that require both speed and accuracy. To limit an opponent’s ability to obtain SA and react, speed is of importance. When team-members or opponents are present, then one must also continuously adapt to a dynamic situation.

Previous research has either focused on more isolated constructs of performance in a static setting (i.e., shoot/do not shoot decisions; Zanesco et al., 2025), or focused on performance metrics for larger-scale team performance in the field (Hancock et al., 2025). This study seeks to fill the gap in measuring individual dismounted soldiers’ performance in ecologically valid room-clearing tasks, which require perceptual-motor skills, including scanning, maneuvering, communicating, aiming and (simulated) shooting. Using a Virtual Reality (VR) system, a set of (portable) room-clearing scenarios are developed, in combination with embedded performance measures. The test is performed individually; although this limits operational realism, it enhances feasibility and consistency for research purposes, as the availability of professional participants is often highly constrained.

Based on input from subject-matter experts and pilot testing (see section 2.3.2.1), three embedded performance metrics were selected and defined: (1) actions performed to obtain necessary situational awareness (SA) of the situation, (2) visual response time (VRT) when shooting armed opponents, and (3) shoot/do not shoot decisions. Obtaining SA is important in CQB, as lethal opponents may be hiding in the rooms to be cleared. The necessary SA is thus obtained by checking blind spots and ensuring that opponents are unarmed or have been eliminated. These actions reflect an operator’s ability to correctly recognize threatening elements in situations, prioritize actions accordingly, and avoid being distracted by irrelevant information. VRT measures the time between the onset of a visual stimulus and the execution of an action and has been identified as a valuable tool for evaluating perceptual-motor performance in tasks requiring rapid responses. For example, research by Heusler and Sutter (2020) demonstrated that tactical police units, compared to patrol officers, exhibited faster VRT and more efficient environmental scanning during high-stress situations. These findings imply that military personnel with advanced training and experience in CQB should similarly display quicker VRTs, reinforcing its potential as a measure of cognitive and psychomotor performance. Whereas VRT reflects the ability to respond quickly when shooting opponents, shoot/do not shoot decisions reflect the ability to make correct decisions when doing so. This metric has previously been used to measure perceptual-motor performance in police and military contexts and is thought to reflect working memory capacity, stress resistance (Scribner, 2016), and inhibitory control (Biggs and Pettijohn, 2022).

To create a simulated interactive operational setting for the current study, a head- and body-mounted Virtual Reality (VR) system was used (Murtinger et al., 2021). Compared to live simulation, VR simulation offers a high degree of control over scenario content, it is relatively cost-effective, and it allows for replaying the scenarios from different viewpoints, including that of the participant. Compared to desktop simulation, VR offers an environment that is more immersive and allows for more realistic interactions with the virtual environment. VR has shown to be effective at providing challenging tasks and inducing stress in military personnel and first responders (Binsch et al., 2022; Linssen et al., 2022; Kleygrewe et al., 2023). In training contexts, VR-enhanced mental skills interventions have been found to produce significantly greater improvements in mental toughness, physiological stress recovery, and decision-making under pressure compared to traditional methods (Gonzalez-Fernandes et al., 2025). These findings indicate that VR is well-suited for both training and assessment of cognitive and psychomotor performance. To evaluate the sensitivity of our test to a military-relevant stressor, one night of sleep deprivation was induced.

This experiment represents a first step towards developing a VR-based assessment tool for evaluating cognitive and psychomotor performance in an operationally relevant context.

We hypothesize that:

1. The embedded measures will demonstrate internal and external reliability, ensuring consistent and valid results across two repetitions of the test.
2. A group of SOF operators will show higher performance than a group of SOF-support personnel, due to more extensive selection and training.
3. The test is sensitive to the negative effects of a night of sleep deprivation. These effects are expected to be more pronounced in the SOF-support group than in the SOF group, as SOF operators undergo more extensive training. Consequently, their professional skills are likely to be more resilient to the impacts of sleep deprivation.

## 2 Methods

### 2.1 Participants

In total, 35 Dutch soldiers (all male, *M* age = 26.0, *SD* = 4.8) participated in this study. The first sample group consisted of 16 participants who worked for a Special Operations Forces (SOF) unit specialized in CQB, *M* age = 28.4, *SD* = 4.3. This squadron is tasked with conducting domestic counter-terrorist operations. The second sample group consisted of 19 participants who worked as SOF-support in the Dutch Army Air Assault Brigade, *M* age = 24.1, *SD* = 4.3. SOF-support soldiers receive specialized training and are qualified to assist the Army’s special operations forces. The participants’ military working experience ranged from 0 to 15 years, SOF *M* = 5.4 years, *SD* = 3.8, SOF-support *M* = 1.5 years, *SD* = 2.5.

For a mixed-model ANOVA, a power analysis using Gpower with alpha = 0.05 and beta = 0.2, correlation = 0.5 and a medium expected repeated-measures and interaction effect size (*f* = 0.25), a total sample size of 34 would be required. For differences between groups, we expected a large effect size (*f* ≈ 0.4), requiring around 40 participants. Any finding for the three dependent measures (SA score, shoot/do not shoot errors, mean VRT) that rejects one of our three hypotheses (see, Introduction) is discussed as potential limitation in validity of the measure concerned. This approach limits risk of family-wise error. Participants gave informed consent before the start of the experiment. The protocol was approved by an accredited medical research ethics committee (MREC Brabant, reference number: P2316, approval number: NL84403.028.23).

### 2.2 Apparatus

**Figure 1:**
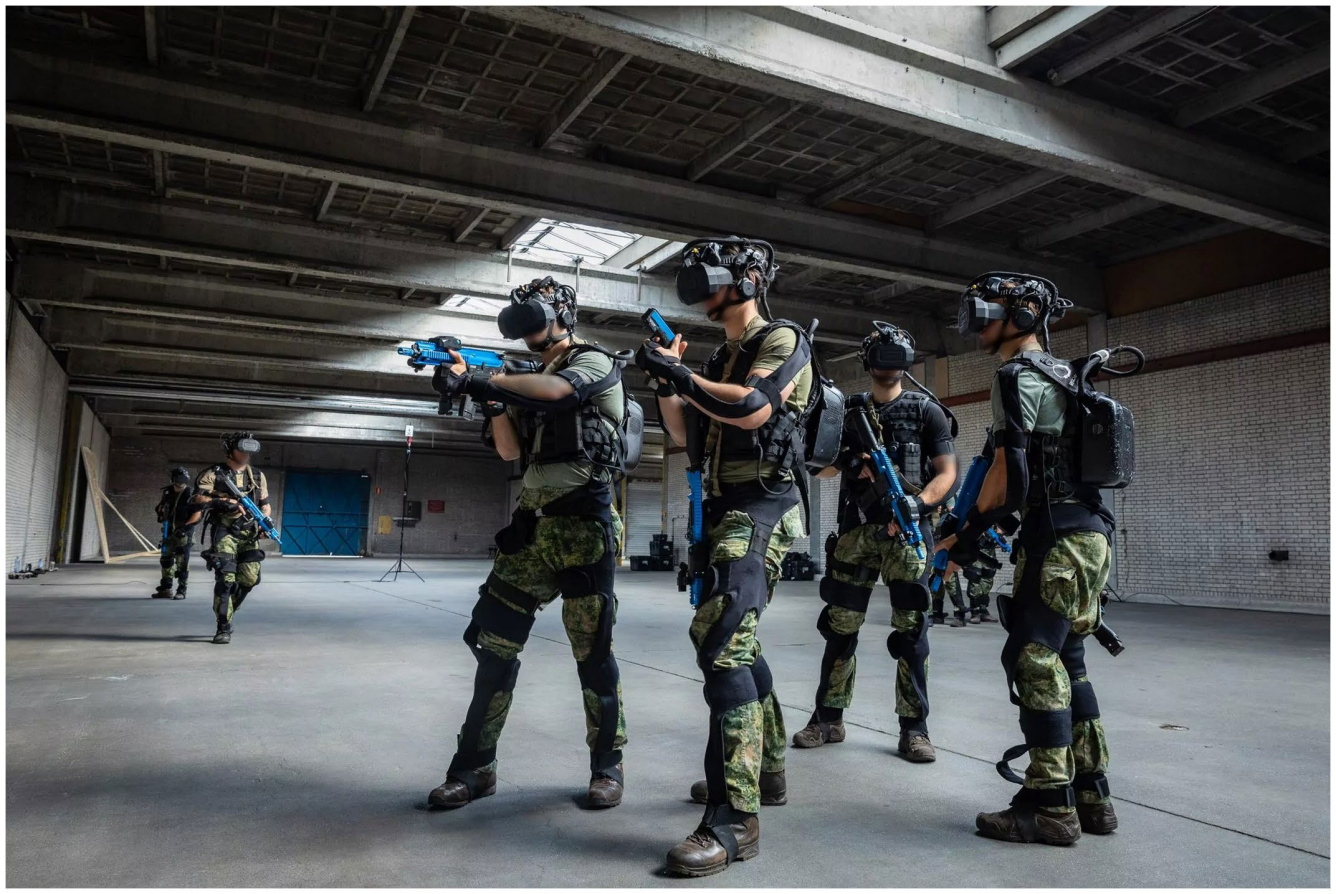
The Blacksuit system used for the VR test. Reproduced from [https://re-lion.com] with permission from the copyright holder.

The Virtual Reality (VR) Blacksuit system (RE-liON©, 2018, Enschede, the Netherlands) has a wireless setup with full-body tracking and head-mounted display (HMD), including a microphone and speakers (see, Figure 1). Participants were equipped with a replica of the Heckler & Koch HK416 and Glock 17, as can be seen in Figure 1. The HMD has a horizontal field of view of 108° degrees and renders 60 frames per second (fps). The system allows for generating a virtual space if 30 by 30 meters and allows participants to see a virtual version of themselves and their weaponry. An airfield hangar of one of the Dutch Airfield Basis was used for the experimental location to conduct the VR test.

### 2.3 Experiment design

Each participant performed a VR test (see, 2.3.2 VR test) once on day one, and once on day two after a night of sleep deprivation. Performance measures were obtained, consisting of dichotomous behavioural SA items (i.e., actions either performed or not), shoot/do not shoot errors, and VRTs (see, 2.4 Dependent measures). Test–retest correlations of these performance measures were obtained (hypothesis 1), and the item-total correlations were obtained for the SA items (hypothesis 1). Then, effects profession group (SOF and SOF-support, hypothesis 2), sleep deprivation (pretest and posttest, hypothesis 3) and the interaction effect were tested using a 2 × 2 mixed-model ANOVA or non-parametric alternatives (see 2.5 Statistical analysis). The study was part of a larger study (see Bottenheft et al., 2024), for which half of the participants received a neurostimulation intervention and half a sham intervention. Effects on performance were found to be non-significant. We therefore did not include this intervention as a covariate in the current design.

#### 2.3.1 Procedure

Participants arrived on day 1 at 6:30 a.m. to receive instructions and provide informed consent. They then put on the full body VR suit. They performed a tutorial to become acquainted with the system and received instructions for the experimental tasks (see, 2.3.2.2 VR test scenarios). The test was then performed individually by two participants at the same time in separate (virtual) rooms, while the rest of the participants waited in a separate (physical) room. Participants left and returned at 7:00 p.m. to receive either active neurostimulation or sham, after which they performed the cognitive desktop tests five times, once every 3 hours (see Bottenheft et al., 2024). After the last test, which ended at 8:45 a.m., they put on the VR suit again and performed a second version of the same VR test scenarios. They then received a present as thanks and were farewelled.

#### 2.3.2 VR test

##### 2.3.2.1 Development and pilot testing

We developed realistic CQB scenarios specifically for the VR test in this study. A pilot test was first performed with a group of 15 SOF operators and 22 regular infantry Marines (Landman et al., 2024). This test was developed with help of two SOF instructors and two SOF operators, as well as three SOF-support CQB experts (Marines). It featured a wider variety of scenario elements and performance measures than the current test, and included situations with multiple unarmed persons in a room, the use of flash bangs, and enemy knife attacks aimed at the participant or at others. Measured performance elements were: communication, manoeuvring to scan for threats, shoot/do not shoot decisions, correctly finishing procedures by checking hostiles who are shot or cuffed, scenario performance speed, and reducing threats by removing firearms or instructing persons to move. Self-reported stress, *M* SOF = 35 on a 100-point scale, *SD* = 22, *M* Marines = 39, *SD* = 19, and mental effort, *M* SOF = 46 on a 150-point scale, *SD* = 17, *M* Marines = 56, *SD* = 22, were reasonable considering that there was no real threat or performance pressure. Stress ratings of the SOF participants in the pilot test did not differ significantly from those in non-virtual SOF training scenarios which involved multiple aggressive actors and paint ammunition (Landman et al., 2024). Based on the participants’ feedback and on exploratory internal and external reliability checks of the performance measures, suitable scenarios elements were selected and expanded upon for the current VR test. This led to a scoping of the current VR test towards the measuring of actions which indicate situational awareness of threats that are induced by blind spots and potentially-armed people (see section 2.4.1).

##### 2.3.2.2 VR test scenarios

**Figure 2:**
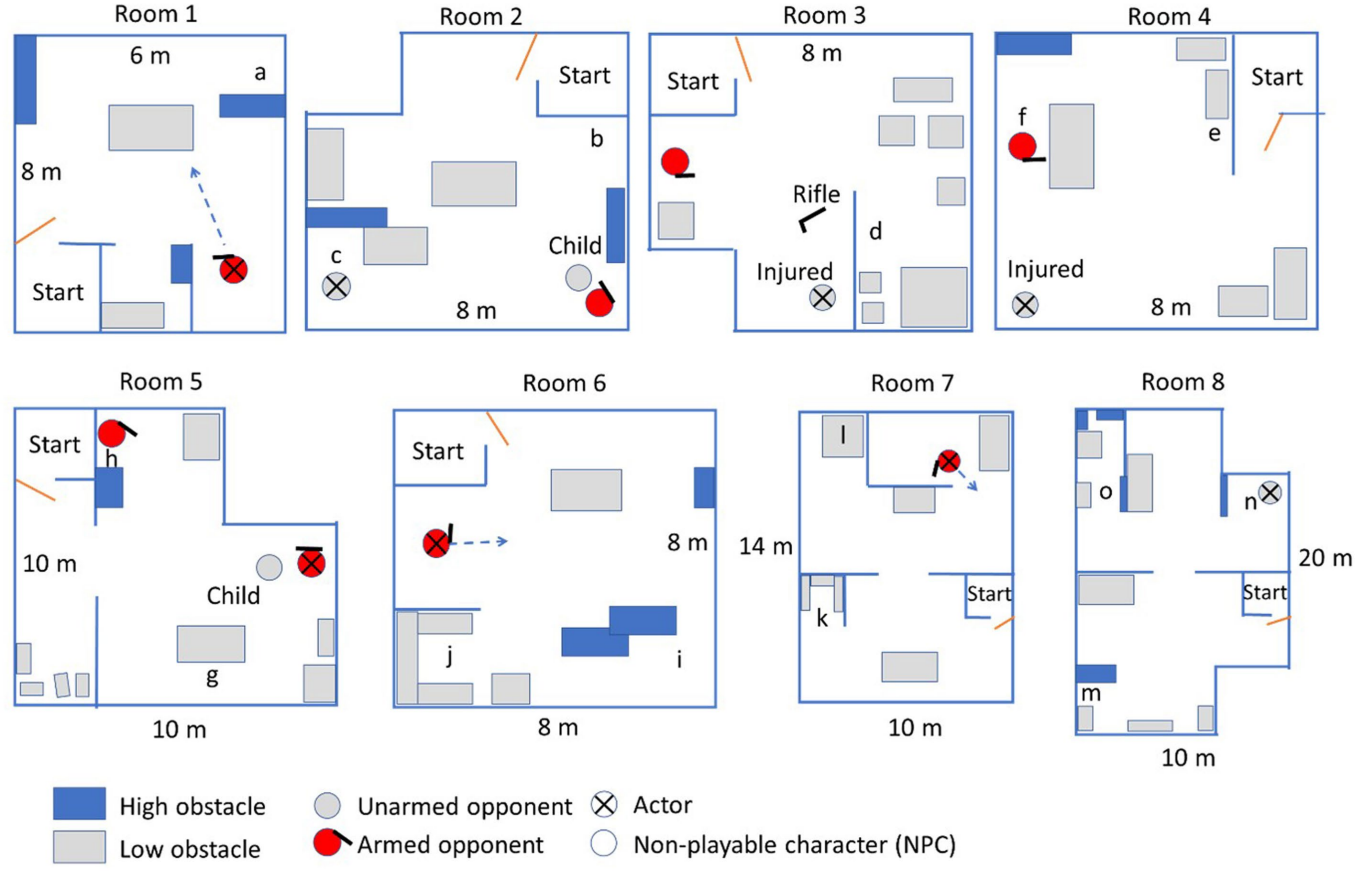
Top view of the eight rooms used in the VR test. Letters indicate locations of blind spots or other elements referred to in Table 1.

**Figure 3:**
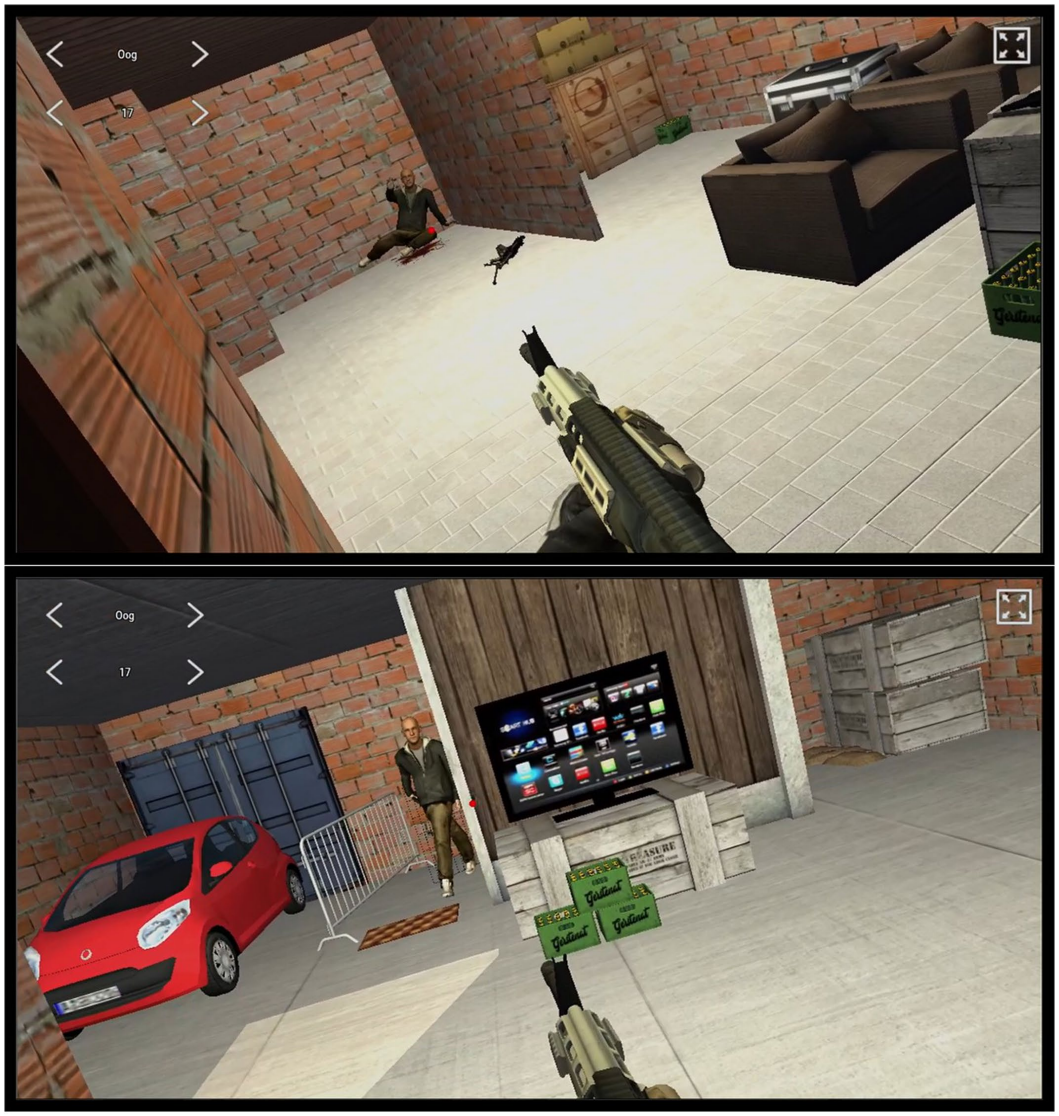
Screenshots of the virtual environment as seen from the participant’s perspective (top: room 3 mirrored, bottom: room 7 mirrored).

The scenarios were performed individually instead of in squad formation, to obtain sufficient statistical power with a limited available number of subjects. The scenarios were designed so that individual performance was possible, and performance measures were limited to aspects of individual performance. The test consisted of eight scenarios, each involving the clearing of one room or two connected rooms (see Figure 2). An actor played one of the people present in the room, whereas other virtual people were controlled from behind a control station. Disruptive noise such as that of a crying child, a barking dog, or a tv playing the news loudly was used in six scenarios. This noise was localized around corresponding objects in the scenario (Figure 3).

For each scenario, participants started in the virtual environment in a separate small room behind a closed door. Participants were instructed that there would be an unknown number of armed opponents and unknown number of unarmed persons in the room. Upon a “start” signal, they were to enter the room and clear it as quickly as possible while ensuring sufficient safety. Rules of engagement stated that any person holding a firearm was to be eliminated immediately. They could not use flash bangs. Making persons in the room kneel and put their hands on their head (instead of cuffing them) was sufficient to eliminate threat. Participants indicated that the room was clear by stating “control”.

For every scenario there was an A and a B version, of which the lay-out was mirrored horizontally. For example, on day one the scenario took place in a household setting, while the second day the scenario was staged in a garage or industrial location with dark walls instead of light walls. In this way, scenarios were comparable enough to test the performance indicators, but different enough to prevent predictability of the required skills or procedures. To prevent systematic influence of the type of scenarios the order of the eight scenarios on each day was counterbalanced between participants using the Latin square method.

### 2.4 Dependent measures

#### 2.4.1 Dichotomous situational awareness items

Table 1 lists the dichotomous situational awareness (SA) items that were obtained in the VR test. The letters mentioned in the item description correspond to the locations shown in Figure 2. The items indicate whether the participant obtained proper SA by scanning blind spots in the room in time (i.e., before walking past them) except for a few items which indicate the obtaining or maintaining of SA by ensuring that hostiles are or remain unarmed (items 3, 14) or eliminated (item 6).

**Table 1:** Dichotomous SA items obtained in the VR test.

Item ID	Room #	Description
1	1	Participant scans blind spot (a) before checking the shot armed opponent.
2	2	Participant scans blind spot (b) when walking to the south.
3	2	Participant makes the unarmed person (c) show their hands before proceeding (actor was instructed to show both hands only after being commanded three times).
4	3	Participant scans blind spot (d) before going to the injured unarmed person or the shot armed opponent.
5	4	Participant scans blind spot (e) before walking into room.
6	4	Participant checks shot armed opponent (f) before helping injured unarmed person.
7	5	Participant first scans behind low obstacle (g) after shooting armed opponent.
8	5	Participant scans behind high obstacle (h).
9	6	Participant scans behind high obstacles (i) after shooting armed opponent.
10	6	Participant scans blind spot (j) before checking armed opponent.
11	7	Participant walks along south wall to check blind spot (k).
12	7	Participant checks blind spot (l) before checking shot armed opponent.
13	8	Participant first checks blind spot (m) before proceeding.
14	8	Participant commands unarmed person (n) to walk out of cover.
15	8	Participant scans blind spot (o) before checking unarmed person.

The letters refer to locations indicated in Figure 2.

#### 2.4.2 Shoot/do not shoot decisions

The number of instances when the participant fired at an unarmed person were counted. Children in the hostage situations were excluded, as these could result from aiming errors. This meant that there was a maximum of four shoot/do not shoot decision errors possible in each test.

#### 2.4.3 Visual response time

**Figure 4:**
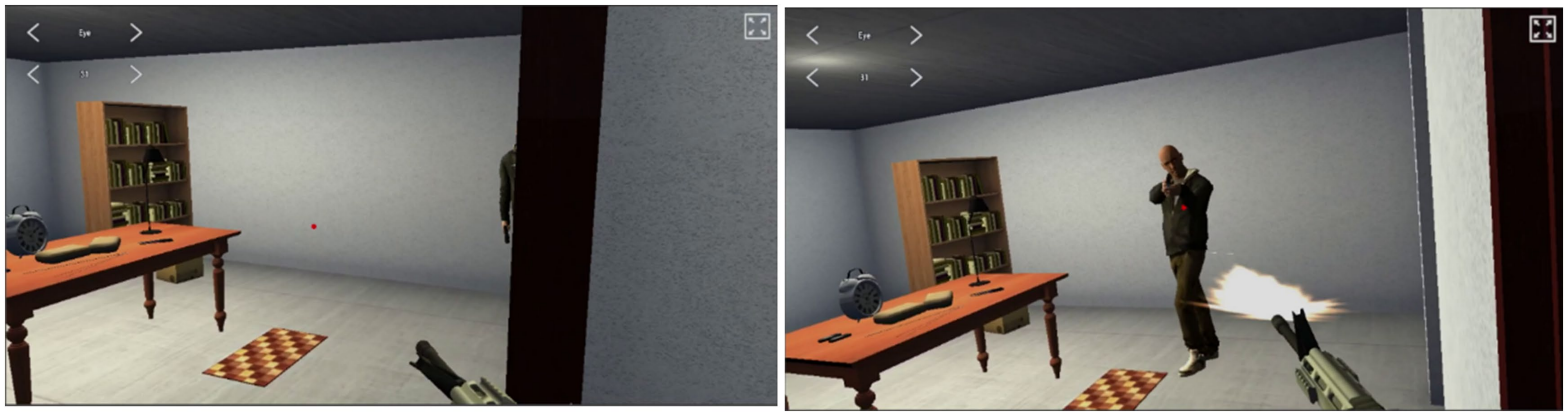
Frames indicating the start (left) and the end (right) of the VRT logged in room 1.

A recording of the visuals as presented through the VR headset to the participant (i.e., their first-person view) was obtained at 60 frames/s and analysed to calculate VRT for 6 predefined shoot actions (room 1, 2, 3, 4, 6, 7) in line with previous research (Nieuwenhuys et al., 2015; Heusler and Sutter, 2020). Room 5 was excluded from this analysis due to one armed opponent squatting behind the hostage child and poor visibility of the other armed opponent due to hiding behind a closet. Frames were counted between the opponent’s firearm appearing into view (Figure 4) and the participant’s first fired shot (Figure 4) using Kinovea video analysis software (kinovea.org) and divided by 60 to obtain VRT in seconds. Outliers (> 2 SDs from total mean) were excluded. The remaining VRTs were combined into one single index by converting VRTs per room to Z scores (i.e., standardizing them) and then taking the mean across rooms.

#### 2.4.4 Motion sickness symptoms

Self-reported symptoms of motion sickness were obtained half-way into the VR pretest and posttest using the Misery Scale (MISC) which ranges from 0 to 10 (Bos et al., 2005). If a participant scored seven (i.e., “medium nausea”) or higher, the VR test was halted, and data was excluded.

### 2.5 Statistical analysis

For determining inter-rater reliability of the 126 SA items (12.7% of data) and 72 VRTs (20.0% of data) were obtained by a second rater. Reliability checks were conducted using Cohen’s Kappa (Landis and Koch, 1977) for the SA items and Intraclass Correlation Coefficients (ICC; Shrout and Fleiss, 1979) for the VRT values. For determining internal reliability, the item-total correlation of each SA item was calculated. SA items with low item-total correlation (*r* < 0.200) in both conditions were removed and Cronbach’s alpha of the remaining items set was obtained for the pretest and posttest. The remaining SA item set was used for further analyses. For external reliability, the test–retest correlation was obtained for the SA items total score and the VRT. For testing our hypotheses with regards to effects of profession group and sleep deprivation, for linear measures (performance items score, VRTs) a mixed-model ANOVA was used with the factors Group (SOF, SOF-support) and Test (pretest, posttest). For ordinal measures (shoot/do not shoot decisions), a Mann Whitney U test was performed to compare performance between groups, and a Wilcoxon Signed Rank test was used to compare performance before and after sleep deprivation. Alpha for significant effects was set to 0.05.

## 3 Results

### 3.1 Internal and external reliability of the VR test measures

There was substantial agreement between the two raters’ judgments for the SA items, *κ* = 0.736, 95% CI [0.625, 0.847], *p* < 0.001. The inter-rater reliability analysis revealed excellent absolute average agreement for the VRTs, ICC = 0.927, 95% CI [0.953, 0.983], *p* < 0.001. Two dichotomous items of the checklist (item 3 and 6; see Table 2) did not meet the criterion of having a correlation of r > 0.200 with the total score in both the pretest and posttest. After removal of these items, Cronbach’s alpha of the remaining item set was 0.69 for the pretest, and 0.78 for the posttest, indicating adequate internal consistency, especially in the posttest. The test–retest correlation of the total score of the remaining SA item set was significant, *r* = 0.428, *p* = 0.015. However, the test–retest correlation of the VRTs was not significant, *r* = 0.262, *p* = 0.147.

**Table 2:** Adherence to each SA item and item-total correlations.

Item ID	Room #	Description	Adherence (%)		Item-total r	
			SOF-support	SOF	pretest	posttest
1	1	Participant scans the north-east blind spot before checking the shot armed opponent.	38.2	40.6	0.582	0.325
2	2	Participant scans the north-east blind spot when walking to the south.	85.3	84.4	0.348	0.300
3*	2	Participant makes the unarmed person (actor) show their hands before proceeding. (Actor was instructed to show both hands only after being commanded three times.)	70.6	90.6	0.076	−0.123
4	3	Participant scans the south-east blind spot before going to the injured unarmed person or the shot armed opponent.	55.9	81.3	0.395	0.489
5	4	Participant scans north-east corner before walking west.	94.1	87.5	0.134	0.264
6*	4	Participant checks shot armed opponent before helping unarmed person.	61.8	53.1	−0.058	0.190
7	5	Participant first scans behind low obstacle after shooting armed opponent.	20.6	16.7	0.151	0.304
8	5	Participant scans behind high obstacle in north-west.	88.3	80.0	0.039	0.300
9	6	Participant walks to north-east after shooting armed opponent to scan behind high obstacles.	23.6	53.3	0.084	0.328
10	6	Participant scans blind spot in south-west before checking armed opponent.	82.4	93.3	−0.005	0.583
11	7	Participant walks along south wall to check blind spot west of starting position.	55.9	83.3	0.680	0.630
12	7	Participant checks blind spot north-west before checking shot armed opponent.	50.0	76.7	0.250	0.567
13	8	Participant first checks blind spot in south-east before proceeding.	47.1	100.0	0.505	0.585
14	8	Participant commands unarmed person to walk out of cover.	20.6	43.3	0.467	0.229
15	8	Participant scans blind spot in north-west before checking unarmed person.	73.5	83.3	0.270	0.475

*SA item was excluded due to poor (*r* < 0.200) item-total correlations.

### 3.2 Effects of profession group and sleep deprivation on SA items total score

**Figure 5:**
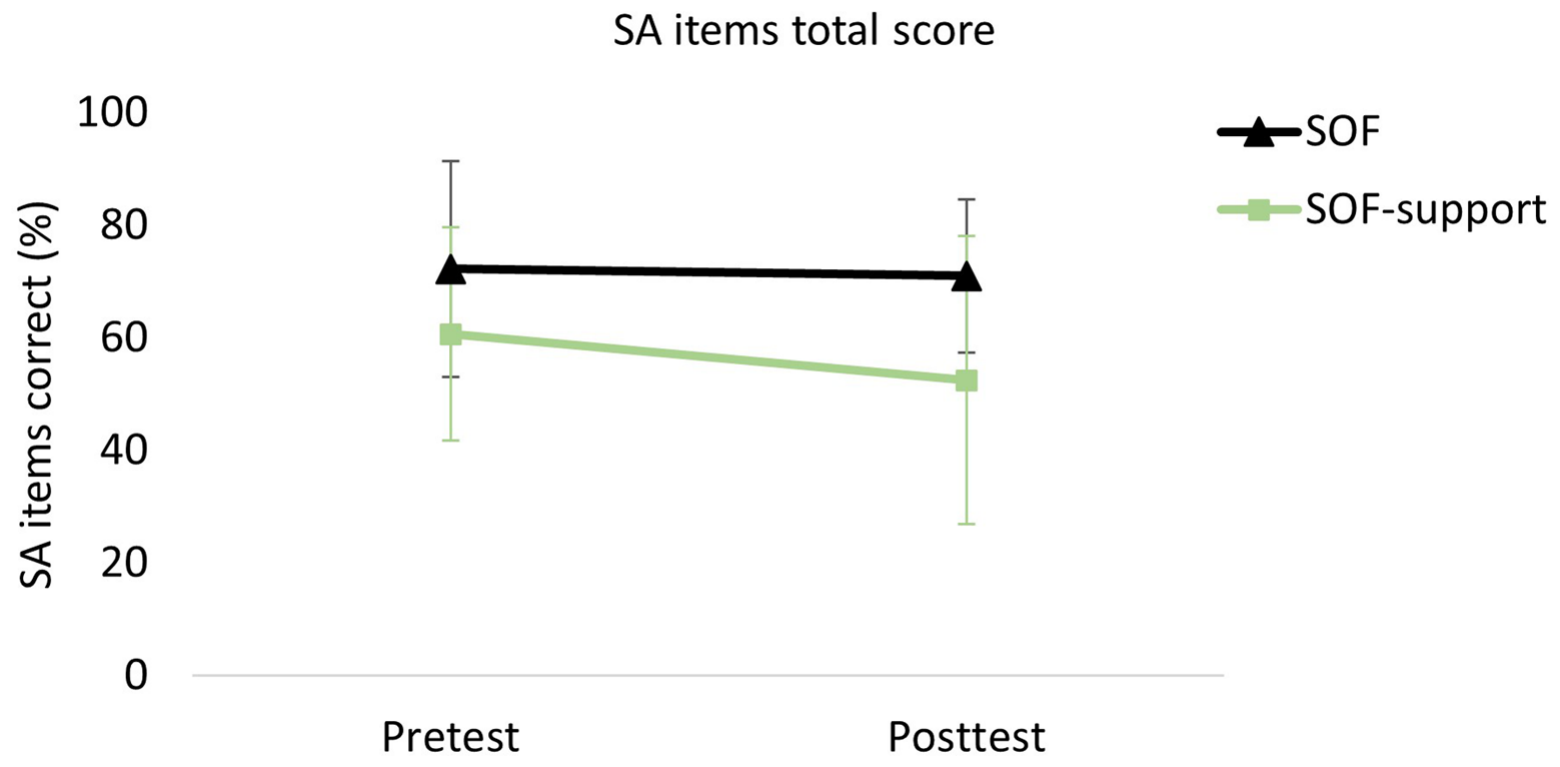
Profile plot of the SA item total score. Error bars indicate SDs.

Figure 5 shows a profile plot of the percentage of SA items total score. There was a significant main effect of Group, *F* (1, 29) = 6.48, *p* = 0.016, but no significant effect of Test, *F* (1, 29) = 0.89, *p* = 0.354, and no significant Group × Test interaction effect, *F* (1, 29) = 0.67, *p* = 0.419. The SOF group, mean = 71.6% correct, *SD* = 16.7%, scored in general significantly higher than the SOF-support group, mean = 56.5% correct, *SD* = 16.7%. The effect size, *η_p_*^2^ = 0.18, was large (i.e., > 0.14).

### 3.3 Effects of profession group and sleep deprivation on VRTs

**Figure 6:**
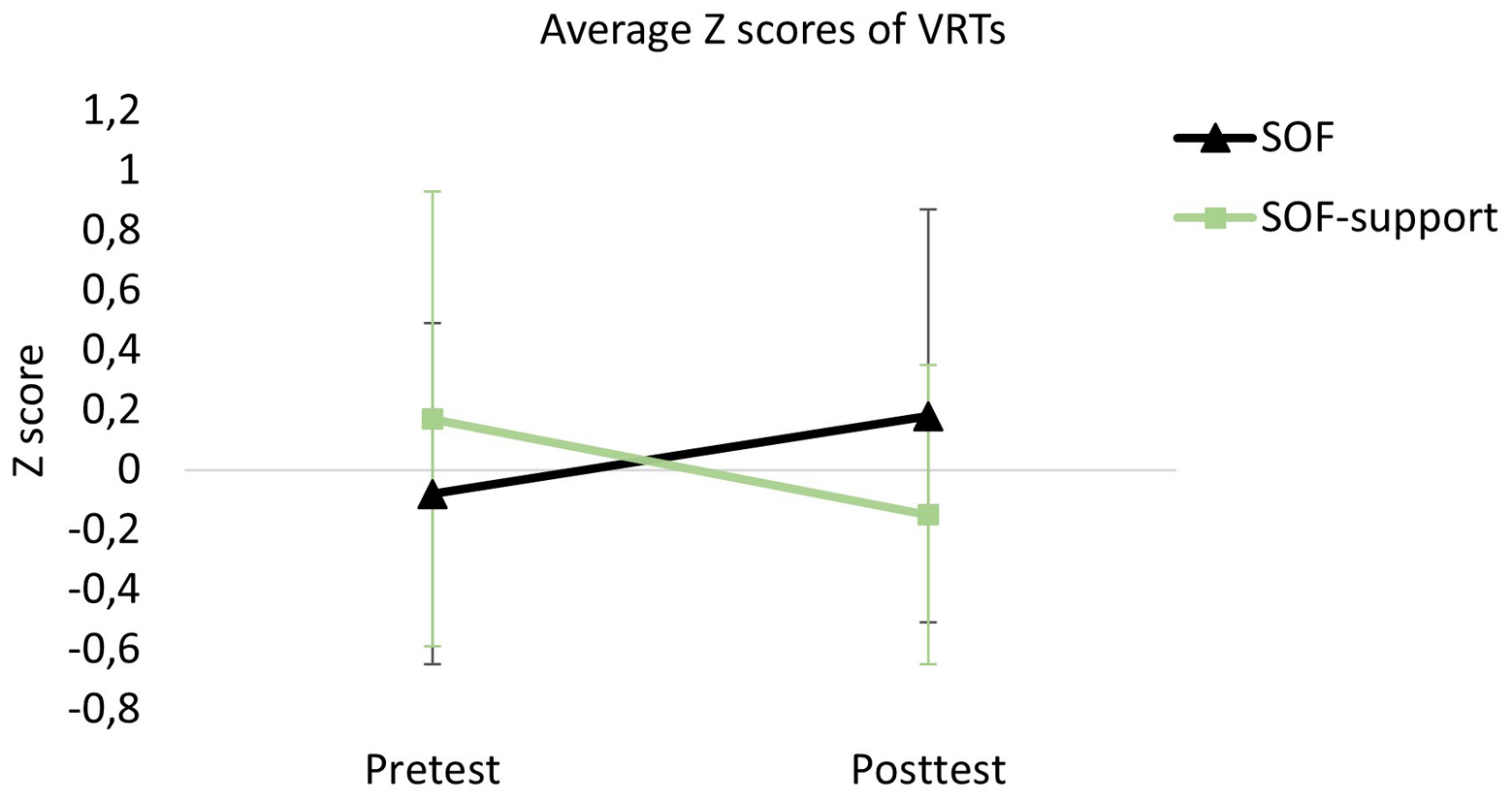
Profile plot of the combined Z scores of the visual response times (VRTs). Error bars indicate SDs.

Descriptive statistics on the VRTs for each group are presented in Table 3. The combined Z scores of the VRTs are shown in Figure 6. There was no significant effect of Group, *F* (1, 30) = 0.24, *p* = 0.629, no significant effect of Test, *F* (1, 30) = 0.06, *p* = 0.812, but there was a significant Group × Test interaction effect, *F* (1, 30) = 4.85, *p* = 0.036. Post-hoc tests on this effect revealed that the SOF-support specialists VRTs marginal significantly decreased between pretest and posttest, *Δ* = 0.32, *p* = 0.085, in the absence of a (marginal) significant difference for the SOF group, *p* = 0.189. There was no significant difference between the groups during the pretest, *p* = 0.306, or the posttest, *p* = 0.133.

**Table 3:** Descriptive statistics of the VRTs of each group in the pretest and posttest.

VRT measure	SOF-support				SOF			
	Pretest		Posttest		Pretest		Posttest	
	*n*	M (SD)	*n*	M (SD)	*n*	M (SD)	*N*	M (SD)
VRT room 1 (s)	14	0.98 ± 0.27	16	0.74 ± 0.16	15	0.98 ± 0.27	14	0.87 ± 0.19
VRT room 2 (s)	10	0.87 ± 0.24	14	0.95 ± 0.29	14	1.03 ± 0.28	14	1.15 ± 0.32
VRT room 3 (s)	15	0.95 ± 0.38	16	0.75 ± 0.36	15	0.76 ± 0.24	15	0.76 ± 0.25
VRT room 4 (s)	14	0.90 ± 0.40	16	0.88 ± 0.23	14	0.77 ± 0.25	15	0.94 ± 0.39
VRT room 6 (s)	15	0.95 ± 0.23	17	0.74 ± 0.27	14	0.79 ± 0.26	14	0.80 ± 0.30
VRT room 7 (s)	15	1.00 ± 0.38	16	1.00 ± 0.45	10	1.09 ± 0.32	10	1.09 ± 0.19
Average Z score	17	0.17 ± 0.76	17	−0.15 ± 0.50	15	−0.08 ± 0.57	15	0.18 ± 0.69

### 3.4 Effects of profession group and sleep deprivation on shoot/do not shoot errors

The Mann Whitney U test showed that the SOF group was significantly less likely to make shoot/do not shoot errors than the SOF-support group, *U* = 68,000, *p* = 0.020. The SOF group made on average 0.27 errors, SD = 0.59, median = 0, whereas the SOF-support group made on average 0.88 errors, SD = 0.81, median = 1. The Wilcoxon Signed Rank test showed that there was no significant effect of sleep deprivation, *Z* = −0.973, *p* = 0.331.

### 3.5 Motion sickness symptoms

Most participants (i.e., 20/35 in pretest, 21/32 in posttest) reported no symptoms of motion sickness (i.e., 0 on the scale). The average MISC score of the included participants was 1.28, SD = 2.02 in the pretest and 1.38, SD = 2.24 in the posttest.

### 3.6 Excluded and lost data

Two participants (one in each group) were instructed to stop the VR test due to their self-reported motion sickness symptoms reaching medium levels of nausea half-way into the pretest. One other participant (SOF-support group) dropped out after the pretest due to an unrelated illness. Their datasets were excluded from analysis. For the remaining 35 participants, 7 action items (0.7% of total data) could not be scored due to scenario errors. Total item scores of the concerning two participants (one in each group) were calculated without these items. A total of 355 VRTs were recorded and 29 VRTs (7.6%) were excluded due to incorrect presentation of the stimulus (e.g., NPC hostile faced the wrong way) or system malfunctions (e.g., participant weapon malfunction). From the remaining VRTs, 13 outliers (> 2 SDs from total mean) were removed.

## 4 Discussion

This paper presents an initial evaluation of the reliability and validity of a test methodology for cognitive and psychomotor performance of dismounted soldiers in an operationally relevant setting. The methodology consists of a set of room-clearing scenarios in a dynamic VR environment, and three performance metrics: (1) actions performed to achieve necessary SA (SA items), (2) shoot/do not shoot errors, and (3) VRTs. We hypothesized that the SA items would demonstrate internal reliability, and that SA items and VRTs would correlate between two moments of testing. Secondly, we hypothesized that a group of SOF operators would perform better than a group of SOF-support soldiers on all three performance measures. Third, we hypothesized that performance measures would be sensitive to the effects of a night of sleep deprivation.

We found partial support for the first hypothesis with regard to the SA items. The inter-rater reliability was satisfactory. With regard to internal consistency, Cronbach’s alpha was 0.69 in the pretest and 0.76 in the posttest. These findings are promising, as Cronbach’s alpha is considered acceptable when ≥ 0.7 (George and Mallery, 2003) and ≥ 0.6 for exploratory research (Hair et al., 2010). The test–retest correlation of the SA items was significant, but still poor, *r* = 0.428 (see, Nunnally and Bernstein, 1994), suggesting some promise as well room for improvement. Individual differences in responses to the neurostimulation intervention or the sleep deprivation may have reduced test–retest correlation. Performance results (see, Figure 5) show that there is no floor- or ceiling-effect for either group, indicating that the test was sufficiently challenging for both groups. These results also show that variation in performance within each group is quite high, and should be further investigated. The requirement to perform the test individually instead of as a team may have led to different approaches between the participants. Observation of outcomes of the separate items (Table 2) indicate that no item appeared too easy (e.g., > 90% adherence) or too difficult (e.g., < 10%) for both groups. Two SA items were excluded from the set due to poor item-total correlations. These items were “making the unarmed person show their hands before proceeding” and “checks shot armed opponent before helping unarmed person”. Perhaps participants regarded these situations less as a threat than was assumed in advance based on opinions of SMEs.

The first hypothesis was not supported for the VRTs, as test–retest correlations were not significant. Also, the variation between participants for the same stimulus was very high (up to nearly 0.5 s, see Table 3). VRT was likely not accurately measured, for several reasons. First, the determination of the onset of the visual stimulus (i.e., the weapon appearing in view) was done through visual inspection, which sometimes led to discrepancies between two observers when a weapon appeared slowly. Second, some participants may have been more careful than others in aiming their first shot to ensure a hit, affecting their VRTs. Third, the limited resolution of the VR system, especially when viewing objects from a distance, may have introduced noise. Lastly, the manner of appearance of the opponent and weapon varied more between participants than was anticipated. Sometimes, the opponent and weapon appeared at the edge of the participants’ field of view, meaning that they first needed to turn their head before responding. Integrated eye-tracking within VR headsets would be needed to address this issue, or the use of more static stimuli (see, Heusler and Sutter, 2020), although this would limit task realism. The development of more advanced synthetic stimuli (e.g., opponents with better artificial intelligence), that are both controlled as well as dynamic could provide a solution for this issue.

The second hypothesis, with regard to group differences, was supported for the SA items as well as for the shoot/do not shoot errors. The results showed that SOF operators had significantly higher SA scores and made significantly fewer shoot/do not shoot errors than SOF-support specialists, supporting validity of these measures. Superior performance on the SA items by SOF may be caused by several factors. First, SOF may possess superior domain knowledge (e.g., “blind spots should always be checked if there is no other immediate threat”) and skills in recognizing threat in situations. Second, they may possess superior innate perceptual-cognitive abilities, allowing them to process visual information more efficiently and effectively (Broadbent et al., 2015; Davids et al., 2006). Lastly the SOF group may have been more adapted at recognizing relevant information and filtering out irrelevant information, enabling them to focus their attention on the task at hand and reducing the mental effort required (Eysenck et al., 2007; Klein, 2005).

In contrast to the significant differences observed in SA scores and shoot/do not shoot errors, there was no significant difference in VRT between SOF and SOF-support specialists. This outcome was anticipated, as the measure did not meet the initial reliability criteria. However, an unexpected but marginally significant interaction effect suggested that the SOF-support specialists exhibited decreased VRTs from pretest to posttest. We propose that this difference may arise from SOF-support specialists being more strictly bound by rules of engagement regarding whether to shoot or not shoot and being less inclined to consider other (possible) actions. As a result, during the second VR test, the SOF-support specialists may have been so predisposed to shoot that they did so more quickly, whereas the SOF group continued to consider alternative courses of action. This tendency toward faster shooting may have been reinforced by participants learning during the test that the avatars often carried firearms. To address this in future research, the test design could introduce more variability by including avatars holding non-threatening objects, such as phones. This would better simulate real-world scenarios, compel participants to observe more carefully to avoid inhibitory errors, and reduce the predictability of the violent nature of the scenarios.

The third hypothesis, regarding the effect of sleep deprivation, was not confirmed by the results. There are several possible explanations for this. First, there may not have been an effect of one night of sleep deprivation on test performance, or this effect was too small to be detected by our measures. Lack of counterbalancing the order of conditions, the timing of the posttest (starting 8:45 a.m. up to 12 p.m. for some), or the type of task (i.e., complex and physically active instead of simple and sitting) may have reduced effects of sleep deprivation. Complex cognitive tasks are less severely affected by sleep deprivation than simple tasks, and performance decrements after sleep deprivation are about twice as severe during circadian night than during day (Wickens et al., 2015).

A limitation of this study is that performance was assessed on an individual level, whereas in real-life, SOF and SOF-support groups always operate in teams. The measures used in this study did not account for aspects of performance related to teamwork and collaborative decision-making. In actual operations, team members must coordinate actions, such as covering each other’s lines of fire when engaging an opponent and adapt to high-pressure situations based on the actions or communication of their teammates. Although this study focused on individual measures, they still provide a strong basis for assessing the impact of selection, training, and human enhancement interventions on various facets of performance related to cognitive and psychomotor performance. This approach contributes to the development of operational evaluation methods for assessing performance. Moving forward, it is recommended that future tests incorporate team performance measures.

In conclusion, the current test methodology showed promising results for evaluating cognitive and psychomotor performance in an operationally relevant and dynamic CQB setting. By integrating embedded measures that capture multiple facets of performance into an ecologically valid assessment, this study underscores the complex nature of operational performance evaluation, moving beyond traditional, simplistic measures. For future directions, our methodology can be applied to other military contexts besides CQB, such as manoeuvring through streets and intersections, and they can be also applied to police tactical units. Future research could include other performance markers, such as shooting accuracy and gaze behaviour. Using our approach, operators’ SA and performance can be measured non-obtrusively using VR, and possibly also in live settings if participants’ positioning in the horizontal plane can be tracked. Algorithmic detection of inappropriate positioning could substitute observational measures, which would increase efficiency and objectivity. Such measures would be highly useful for providing feedback for training purposes, or for obtaining data for research purposes. The developed methodology therefore facilitates the evaluation of human enhancement interventions on cognitive performance in realistic and operationally relevant settings.

## Data Availability

The datasets presented in this article are not readily available because of confidentiality considerations concerning the participant group. Access to the data is restricted to ensure the confidentiality and protection of the individuals involved. Requests to access the datasets should be directed to matthijs.koedijk@tno.nl.
